# An Electrochemical Glucose Sensor Based on Zinc Oxide Nanorods

**DOI:** 10.3390/s150818714

**Published:** 2015-07-30

**Authors:** Mohammed Marie, Sanghamitra Mandal, Omar Manasreh

**Affiliations:** 1 Microelectronics and Photonics graduate program, University of Arkansas, Fayetteville, AR 72701, USA; 2 Department of Electrical Engineering, University of Arkansas, Fayetteville, AR 72701, USA; E-Mails: sm009@uark.edu (S.M.); manasreh@uark.edu (O.M.)

**Keywords:** electrochemical sensor, glucose, zinc oxide nanorods, electrode

## Abstract

A glucose electrochemical sensor based on zinc oxide (ZnO) nanorods was investigated. The hydrothermal sol–gel growth method was utilized to grow ZnO nanorods on indium tin oxide-coated glass substrates. The total active area of the working electrode was 0.3 × 0.3 cm^2^ where titanium metal was deposited to enhance the contact. Well aligned hexagonal structured ZnO nanorods with a diameter from 68 to 116 nm were obtained. The excitonic peak obtained from the absorbance spectroscopy was observed at ~370 nm. The dominant peak of Raman spectroscopy measurement was at 440 cm^−1^, matching with the lattice vibration of ZnO. The uniform distribution of the GO_x_ and Nafion membrane that has been done using spin coating technique at 4000 rotations per minute helps in enhancing the ion exchange and increasing the sensitivity of the fabricated electrochemical sensor. The amperometric response of the fabricated electrochemical sensor was 3 s. The obtained sensitivity of the fabricated ZnO electrochemical sensor was 10.911 mA/mM·cm^2^ and the lower limit of detection was 0.22 µM.

## 1. Introduction

Sensing glucose by using ZnO nanorods is an inexpensive, safe, accurate, fast, and harmless approach [1,2]. The nanostructure of this semiconductor material has several optical, chemical, and electrical properties that make ZnO one of the best candidates used in electrochemical sensors. For example, it has a wide band gap that makes it a stable semiconductor in the visible region and it can be synthesized using sol – gel growth method [3,4,5]. Zinc oxide is a biocompatible material suitable for easily medical and biological applications such as wearable devices. The high ability to react with oxygen makes ZnO one of the best potential semiconductors materials to be used in electrochemical sensors fabrications [6]. The operation of the zinc oxide nanorods electrochemical sensor is based on an electrochemical reaction between ZnO nanorods and the electroactive species in the blood such us glucose. The output signal that can be detected is an electric current and this is through several steps [7,8]. Glucose oxidase is a stable enzyme, which helps in oxidizing glucose to glucolactone and to convert the oxygen into hydrogen peroxide. Hydrogen peroxide is a simple compound consisting of a single oxygen–oxygen bond [9]. It is a colorless compound if it is a pure solution, and it has a moderately higher viscosity than water. The second significant reaction in the sensing mechanism is the reaction between the enzyme and oxygen. This generates the hydrogen peroxide, and the ratio of hydrogen peroxide can be detected by the working electrode [10]. The final step of the reaction is the production of free electrons. This step is known as the dissociation of hydrogen peroxide, and the free electrons are sensed by the sensor as an output electric current [11].

In this paper, we report on a cost effective, accurate, fast, and highly sensitive electrochemical sensor based on ZnO nanorods and a sol–gel technique. A titanium layer with 100 nm thickness is used to form the contact area on top of the ITO in the working electrode. The total area of the working electrode is 0.3 × 0.3 cm^2^ and the reference electrode, which is made of platinum, has the same area. Glucose oxidase is spin coated on top of the working electrode and left in air for two hours to give more opportunity for the ZnO nanorods to absorb the GO_x_. A thin membrane of Nafion is also spin coated to prevent any enzyme leakage and give more stability to the working electrode. The hexagonal structure of the as grown ZnO nanorods was determined by using scanning electron microscopy (SEM). Sensitivity, lower limit of detection, and glucose reduction, depend mainly on the alignment of the as grown ZnO nanorods and the uniform distribution of their diameters. The as grown ZnO nanorods are also characterized by using absorbance and Raman spectrum after annealing in 150 °C.

## 2. Experimental Section

### 2.1. Seed Layer Growth

Among several fabrication approaches, hydrothermal growth is an effective chemical method to successfully synthesize ZnO nanorods. It is simple, cost effective, and low growth temperature technique. There are some other low temperature techniques to synthesis ZnO nanorods, such as the sonochemical method, but it is not easy to control the dimensions of the synthesized nanorods. In addition, since it is a solution-based method, the pH of the solution must be fixed at a certain value using an expandable ion analyzer, and this adds more complexity to the entire technique. The aspect ratio, which is the ratio between the length of the as grown ZnO nanorods and their diameters, plays an important role in the performance of the fabricated sensor, and this can be controlled using the hydrothermal method since the growth time and temperature determine the length of the synthesized ZnO nanorods [12]. For high quality material, the seed layer plays the most significant role in order to determine the morphology of the ZnO nanorods. To prepare this precursor, zinc acetate dihydrate (Zn(CH_3_COOH)_2_·2H_2_O) at 0.5 M was mixed with 10 mL of methoxyethanol (CH_3_H_8_O_2_ 99%) at 75 °C and stirred at 300 rotations per minute (RPM) for an hour. A small volume of ethanolamine (0.3 mL) is added as a mediator to prepare the seed layer. After dissolving the zinc acetate dihydrate in the methoxyethanol, the seed layer solution is placed under ultrasound for an hour to increase the homogeneity of the precursor. Even if the precursor was stirred with high speed (300) RPM for one hour and placed in the ultrasound for another hour, still some big particles don’t dissolve completely and because of that fact, a small filter is used in this process [10].

### 2.2. Growth Solution

**Figure 1:**
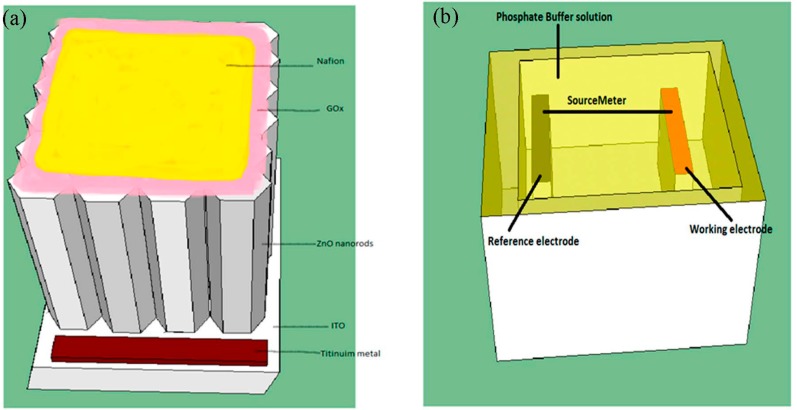
The structure of the working electrode (**a**), and the schematic structure of the electrochemical experimental setup (**b**).

The second major step is synthesizing the growth solution. 0.025 M zinc nitrite (Zn(NO_3_)_2_·6H_2_O) and hexamethylenetetramine (0.025 M) are each dissolved in 10 mL of deionized water in two separate containers. The two prepared precursors are stirred for one hour separately at 300 RPM at room temperature and then mixed together very slowly drop by drop using a micropipette [11]. The prepared solution has a very short lifetime, so it should be used immediately and the homogeneity of the growth solution affects the final shape of the grown as nanorods. The growth time varies from three to six hours depending on the desired length and diameter of the as grown nanorods. Figure 1a illustrates the working electrode coated with glucose oxidase and a nafion membrane to increase the sensitivity and decrease the lower limit of detection, and Figure 1b in the same figure shows the electrochemical experimental setup.

## 3. Results and Discussions

**Figure 2:**
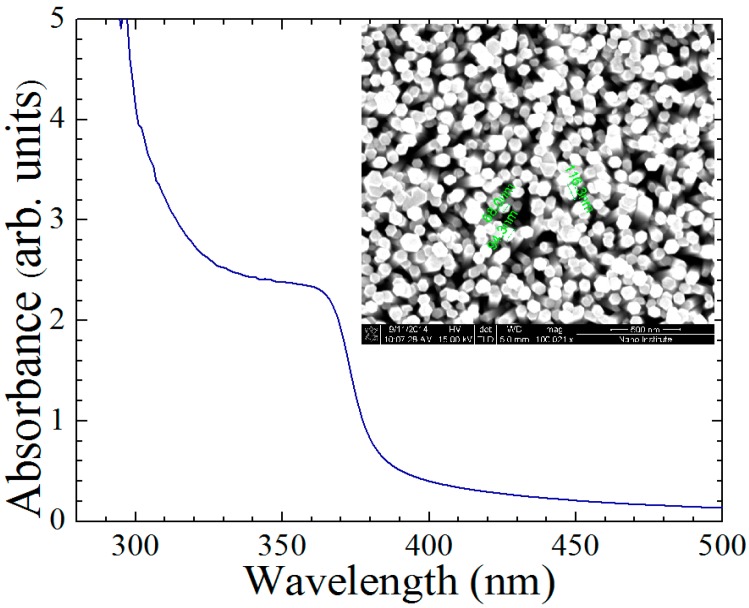
Absorbance spectrum of ZnO nanorods and its optical peak is around 370 nm corresponding with the band gap of the material and the inset figure refers to the hexagonal structure of the ZnO nanorods that was taken by SEM image with a variety in diameter from 68 to 116.3 nm.

The absorbance spectrum of the prepared ZnO nanorods after annealing at 150 °C is shown in Figure 2. The effect of annealing at 150 °C helps in reducing the non-radiative crystal defects and helps in enhancing the optical absorbance. Annealing at 150 °C increases the grain size and results in single crystal ZnO nanorods, which is a desirable crystal structure for use in the electrochemical sensor. The optical excitonic peak can be clearly seen with a wavelength ~370 nm and, it corresponds to the direct band gap of the ZnO, 3.37 eV. The optical absorbance of the ZnO nanorods increased rapidly after the energy of the incident photons became higher than the band gap. This is an indicator that the ZnO semiconductor absorbs the light at the edge of the visible region. Working at the edge of the visible region gives high stability and less noise to devices based on the ZnO nanostructure. The hexagonal structure of the as grown ZnO nanorods is shown in the inset in the same figure. Depositing three layers of the sol–gel with five minutes drying on a hot plate at 110 °C for each layer ensures high adherence to the seed layer on top of the ITO, and as a result, the as grown ZnO nanorods were well aligned with an acceptable variety in their diameters from 68 to 116.3 nm.

**Figure 3:**
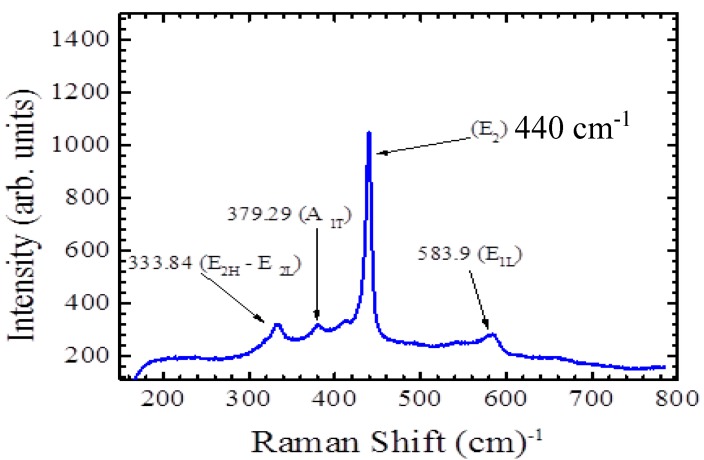
Raman spectrum of the grown ZnO nanorods and the observed peaks that correspond to the phonon vibration at room temperature.

The lattice and phonon vibration modes are shown in Figure 3. Raman spectroscopy provides information regarding the lattice vibration of nanostructure materials. The peak that belongs to the ZnO lattice vibration at 440 cm^−1^ appears to be sharp and narrow. The other small peak on the right at 575 cm^−1^ corresponds to the oxygen vaccines in ZnO nanorods. To obtain deeper information by using Raman spectroscopy, it is much better to characterize ZnO nanorods at low temperatures around 10 K. The ratio of zinc and oxygen in ZnO nanorods is clearer at low temperatures and those small peaks on the left 340, 375, and 400 cm^−1^ can be analyzed accurately. According to quantum mechanics, reducing the temperature provides information regarding the natural lattice vibration that occurs at the zero point energy, so all the possible phonon and lattice modes represent the actual vibrations of the ZnO nanorods.

**Figure 4:**
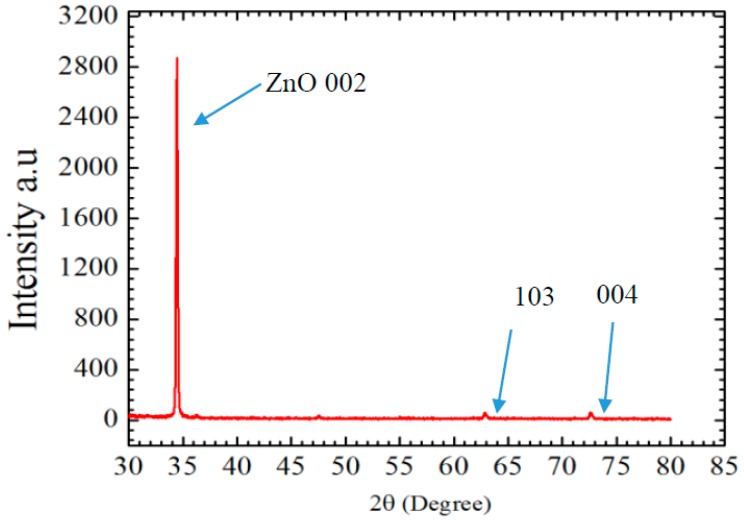
XRD pattern of ZnO nanorods after annealing at 150 °C.

X-ray (XRD) diffraction analysis is performed using Phillips X-ray diffractometer with wavelength 1.54 Å to determine the purity of the material and to calculate the grain size of synthesized ZnO nanorods. Figure 4 shows the XRD pattern of the material and the peak that corresponds to ZnO appears at 34.364°. Miller indices for the observed peak were 002, andthe grain size of ZnO nanorods was calculated by using Scherrer equation [13]:

$$\frac{K\lambda }{\text{β}\;cos\text{θ}}=G\;$$

where G is the grain size in A°, K is Scherrer constant and its value is around 0.9, λ is the wavelength of the X-rays, β is the full width at the half maximum of the peak, and θ is the angle that corresponds with the observed peak. The grain size is calculated using the above equation and it was around 1.6 nm. It is an indicator that the synthesized ZnO nanorods are single crystals. The other small peaks occur at 62.756 and 72.429 respectively and they belong to Miller indices 103 and 004.

**Figure 5:**
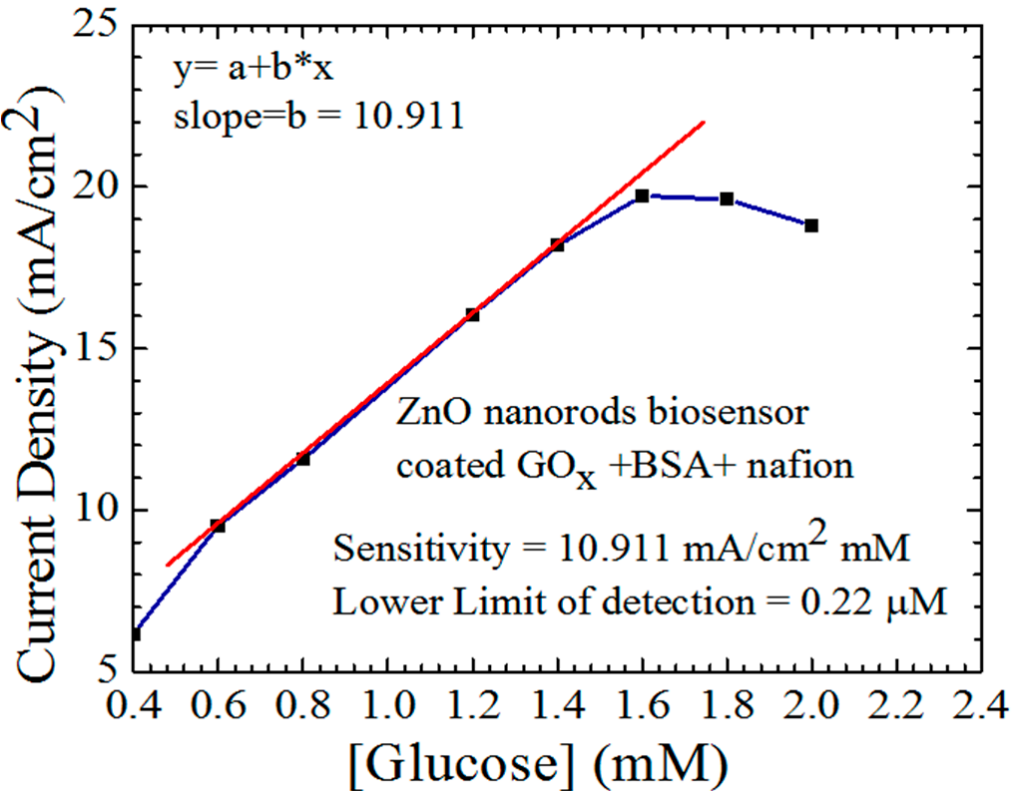
The sensitivity and the lower limit of detection of the fabricated ZnO nanorods electrochemical sensor were calculated from the linear line that starts from 0.6 to 1.4 mM.

The sensitivity of the fabricated ZnO nanorods electrochemical sensor is shown in Figure 5. The measurement was performed by using a Keithley 2410 SourceMeter. Platinum rod was used as a reference electrode, and both working and reference electrodes were immersed in a pH 7 phosphate buffer solution. The measurement started with zero mM glucose concentration, and the glucose was added continually starting from 0.4 mM up to 2.4 mM. The sensitivity in this test is the slope of the linear line that starts from 0.6 to 1.4 mM. The response of the sensor to the changes in the glucose concentrations can be represented by the straight increase in the output current until it reaches the saturation point at 1.6 mM. The lower limit of detection, which is the lowest concentration of glucose that can be detected by the device, can be calculated by using the same figure. The formula to calculate the lower limit of detection is 3×σ/slope, where σ is the standard deviation of the current density, and it was found to be 0.22 μM, which means that the sensor is sensitive to lower concentrations of glucose.

**Figure 6:**
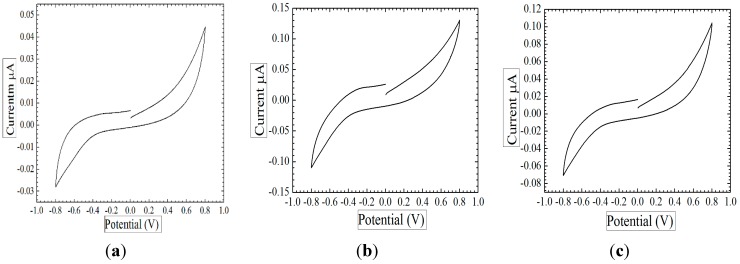
The oxidation reduction of glucose at the peak of 0.8 V is illustrated in the cyclic voltammetry curve of the ZnO nanorods electrochemical sensor where (**a**) is in the absence of glucose; (**b**) is at 1 mM glucose concentration; and (**c**) is at 2 mM glucose concentration.

Oxygen reduction is one of the most significant mechanisms that provide useful information about the oxygen limit of the ZnO nanorods electrochemical sensor. The measurement was done using the potentiostat method. The first measurement was performed by connecting the working and reference electrodes to the potentiosat while they are immersed in a pure phosphate buffer without glucose. It can be noted that the sensor showed a low response in the absence of glucose. The maximum output current was 0.045 μA, as shown in Figure 6a, which is lower than the current at the presence of glucose at 1 mM and 2 mM. The same measurement was repeated with 1 mM glucose concentration and 2 mM glucose concentration. The process starts when the oxygen level is stable and no reduction process occurs with zero current. By changing the potential of the working electrode, the current starts increasing slowly with oxygen reduction. When the current reaches its maximum level at 0.8 V, which is the same voltage that was used to measure the sensitivity, the potential of the working electrode is reversed by changing the sweep direction. During this reversal, the chemical reaction that took place between the working electrode and the oxygen can be investigated as a peak corresponding with a certain working electrode potential. The maximum output current of the oxidation reduction process at 1 mM glucose concentration was 0.11 μA at 0.8 V as it is shown in Figure 6b of the same figure. The sensor exhibited high sensitivity toward changes in glucose concentration, and the output current was 0.125 µA in the presence of 2 mM of glucose at the same potential, and this can be found in Figure 6c. It is also a way to indicate the potential of the working electrode and to avoid going beyond it.

**Figure 7:**
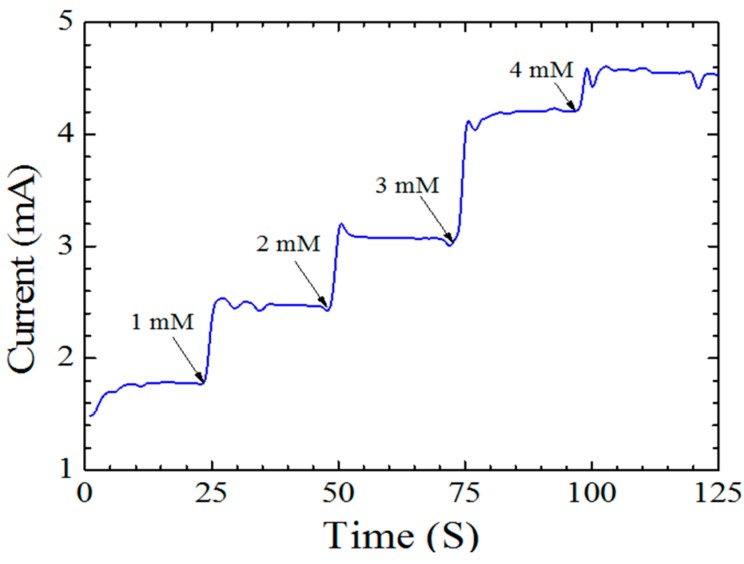
Amperometric response of the fabricated electrochemical sensor to different concentration of glucose with 3 s response time.

The current *versus* time response is illustrated in Figure 7. This test is performed by using the SourceMeter 2410 with 0.8 V applied voltage. The glucose was added to the phosphate buffer continually after each 25 s. The observed ZnO nanorods electrochemical sensor response time is 3 s, which can be considered a very short time. It gives information about how fast the response of the fabricated ZnO sensor is to any small changes in the concentration of glucose. The horizontal axis is the time and the length of the horizontal intervals is very significant. The shorter the interval means the higher the response of the device and *vice versa*. This test is done with different concentrations of glucose starting with 1 mM and ending with 4 mM with the presence of the GO_x_ and Nafion membrane. The GO_x_ works a mediator and the Nafion membrane helps in increasing ion exchange and prevents any enzyme leakage. It also provides a better isolation to the working electrode to prevent any external reaction that might increase the noise, the lower limit of detection and decrease the sensitivity.

In the inset of Figure 2 the well aligned ZnO nanorods with a uniform distribution in their diameter, appear to have high surface to bulk ratio and hexagonal structure. This reflects the effectiveness of the sol–gel technique used [14]. Another result was published regarding the growth of ZnO nanorods. The authors in that case obtained ZnO nanorods at high temperatures starting from 350 to 500 °C. Despite the use of high temperatures, the alignment is still a problem even though it improved with increasing temperature. In this work, well aligned ZnO nanorods were obtained at 150 °C which is not considered a high temperature [10]. The uniform distribution of the ZnO nanorods and the good alignment help in increasing the absorption of oxygen and consequently, resulted in a glucose sensor with high output despite the lower glucose concentration level [15]. The time response of the ZnO nanorods electrochemical sensor, as shown in Figure 7, was 3 s and it is considered a short time. It indicates that the current reaches 95% of the steady state value in only 3 s. This is an indicator of the fast electron exchange between the working electrode and the phosphate buffer solution (PBS). It is considered a fast response compared with other ZnO nanorods electrochemical sensors [16]. The measurements were performed with different concentrations of glucose starting with 1 mM and ending with 4 mM. The interval time was 25 s between each measurement and from this test, the sensitivity of the fabricated device can be predicted because the measurement refers to how sensitive the sensor is to any small changes in the glucose concentration [17]. The amperometric response of the fabricated sensor can be increased by increasing the ion exchange through the nafion membrane and the uniform distribution of the glucose oxidase as a sensing material [18].

In Figure 3 the lattice thermal vibration that corresponds to the wave number 575 cm^−1^, which belongs to ZnO nanorods at room temperature is invistigated. The peak that represents the phonon vibration that comes from the thermal lattice vibration is sharper than the reported Raman spectroscopy of ZnO nanorods measurement [19]. The other two small peaks on the left belong to the other component of the ZnO nanorods. Different ratios of oxygen in the ZnO compound might produce different lattice vibrations, so the Raman spectroscopy results in this report is more precise than in some reported papers and that could be the effect of annealing at 150 °C [20]. Another factor might be the substrate since the ZnO nanorods grown on top of silicon gave different peaks that are not sharp and precise and the other small peaks were not clear at all [9]. The sensitivity of the electrochemical sensor was measured by investigating the relationship between the changes in the current and the changes in the glucose concentrations and this can be seen in Figure 5. It was measured by calculating the slope of the straight line that starts from 0.6 and ends at 1.4 mM. The sensitivity of the glucose electrochemical sensor was 10.911 mA/mM·cm^2^, which is higher than the sensitivity of glucose electrochemical sensors fabricated and reported in previous studies [21]. The reported lower limit of detection in this current work is 0.22 μM, which is less than the reported lower limit of detection of other ZnO electrochemical sensors.

## 4. Conclusions

An electrochemical glucose sensor based on ZnO nanorods was conceived, fabricated and tested. The high quality of the grown ZnO nanorods characterized by SEM and the absorbance spectrum contributed significantly in enhancing the sensitivity and reducing the lower limit of detection. The high sensitivity of the sensor is also confirmed by the amperometric test that exhibits a quite short time response around 3 s for any change in glucose concentration. Spin coating the glucose oxide on top of the ZnO nanorods helps in reducing the bias voltage of the working electrode and enhances the sensitivity of the fabricated electrochemical sensor. The ion exchange between the phosphate buffer solution and the ZnO nanorods was performed properly by spin coating a nafion membrane on top of the glucose oxidase. This nafion membrane helps in reducing the enzyme leakage and increases the stability of the device by preventing undesired chemical reactions that might occur during the electrochemical processes.
